# *Hedychiumziroense* (Zingiberaceae), a new species of ginger lily from Northeast India

**DOI:** 10.3897/phytokeys.117.24951

**Published:** 2019-02-06

**Authors:** Ajith Ashokan, Vinita Gowda

**Affiliations:** 1 Tropical Ecology and Evolution (TrEE) Lab, Department of Biological Sciences, Indian Institute of Science Education and Research (IISER) Bhopal, Bhopal, 462066, India Indian Institute of Science Education and Research Bhopal India

**Keywords:** Apatani, Arunachal Pradesh, *
Hedychium
*, taxonomy, Ziro

## Abstract

We describe *Hedychiumziroense***sp. nov.** from Northeast India (NE India) which was discovered during one of our recent botanical explorations in Arunachal Pradesh. We provide detailed morphological comparison of this species with four other *Hedychium* species (*H.griersonianum* R.M.Sm., *H.ellipticum* Buch.-Ham. ex Sm., *H.gomezianum* Wall. and *H.yunnanense* Gagnep.), with which it shares some morphological similarities. The new species is characterised by a dense cylindrical spike, pubescent rachis, folded bracts, 2–3 flowers per cincinnus, deeply cleft labellum and a distinctive late monsoonal flowering phenology from August to September.

## Introduction

The genus *Hedychium*, comprising more than 80 species (Sanoj 2011), is distributed in the tropical, subtropical and warm-temperate Asia (Indian subcontinent, China and Southeast Asia) and Madagascar (Sirirugsa 1991, Newman et al. 2004, Newman and Pullan 2007, Tanaka et al. 2016, Ding et al. 2018, WCSP 2018). Based on inflorescence type and bract structure, Wallich (1853) recognised four subgeneric divisions within *Hedychium*: *Coronariae* (spikes with more or less tightly imbricate bracts), *Spicatae* (spikes with distant, spreading bracts), *Siphonium* (characterised by stemless habit and crested anther) and *Brachychilum* (characterised by an exceedingly small labellum). Based on the relative lengths of stamen and labellum, Horaninow (1862) divided the genus into three groups: *Gandasulium* (species with shorter stamen compared to labellum), *Macrostemium* (species with stamen length equal to or exceeding the labellum length) and *Brachychilum*. Later, Baker (1892) treated *Gandasulium* (to be corrected as *Hedychium*, as it includes the type of the genus, M. Newman pers. com.) and *Macrostemium* as two different sections within the genus *Hedychium*. Finally, based on the shape of the inflorescence and concealment of the inflorescence rachis, Schumann (1904) divided *Hedychium* into two subgenera: *Gandasulium* (to be corrected as *Hedychium*, mentioned above; characterised by wide, ellipsoid or ovoid inflorescence, dense imbricate bracts and hidden rachis) and *Eosmianthus* (characterised by longer than wide inflorescence, spreading bracts and exposed rachis). However, the molecular phylogeny of *Hedychium* by Wood et al. (2000) revealed that the morphologies referred to by Wallich (1853), Horaninow (1862), Baker (1892) and Schumann (1904) were not synapomorphic with any of the subgeneric groups and therefore the subgeneric groups were not supported. Instead, four distinct clades were now identified in the phylogenetic tree based on their biogeographic pattern; clade 1 - mostly Peninsular Malaysian taxa, clade 2 - Chinese and high elevation Himalayan taxa, clade 3 – *Hedychiumacuminatum* and clade 4 - mostly Indian and Burmese (Myanmar) taxa. Interestingly, the number of flowers per cincinnus was the only morphological character that correlated with the clades wherein clade 2 was represented only by the species with 1-flowered cincinnus.

In India, *Hedychium* is the most diverse genus in the family Zingiberaceae and it is represented by at least 44 taxa (31 species and 13 varieties) from NE India and the Western Ghats (Sanoj 2011). Taxonomic revisions and explorations in the last few decades have resulted in the discovery of several new *Hedychium* (species as well as varieties) from the NE India and the Western Ghats (Sanoj et al. 2010, Sanoj and Sabu 2011, Thomas et al. 2015, Odyuo and Roy 2017). However, the total species count of *Hedychium* in India remains ambiguous due to the lack of detailed taxonomic studies, incomplete geographical sampling caused by difficult terrain and political disturbances (Srivastava 1984, Sarangthem et al. 2013, Sarangthem 2015, Ashokan and Gowda 2017). Northeast India consists of eight states, out of which Arunachal Pradesh (previously known as NEFA or North-East Frontier Agency) holds a major share of the Eastern Himalayan biodiversity hotspot (Mani 1974, Paul et al. 2005). It is second highest (20 species) in *Hedychium* diversity in India, next to Meghalaya (28 species, pers. obs.). Three *Hedychium* species (*H.radiatum*, *H.raoii* and *H.robustum*) are known to be narrow endemics to Arunachal Pradesh and they are known only from their respective type localities (Rao and Hajra 1974, Pal and Giri 1998, Sanoj 2011).

**Figure 1.**
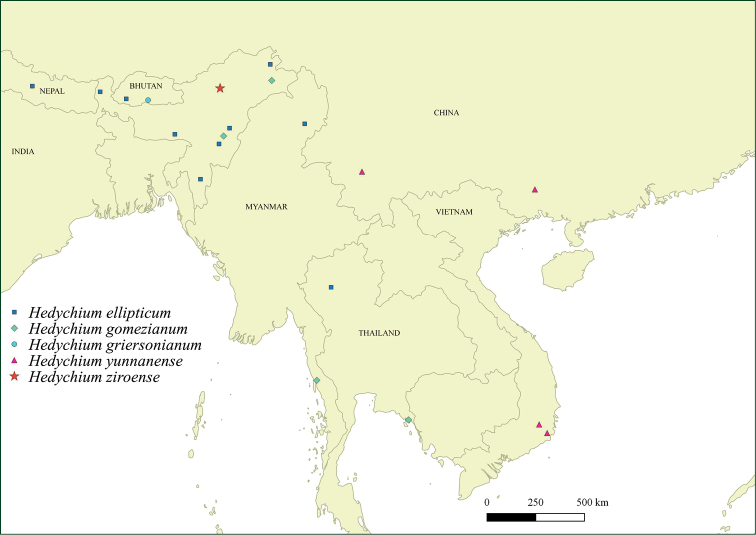
Distribution of *Hedychiumellipticum*, *H.gomezianum*, *H.griersonianum*, *H.yunnanense* and *H.ziroense* sp. nov. in the Indian subcontinent, China and Southeast Asia.

Here we describe a new species of *Hedychium*, characterised by more than 1-flowered cincinnus in each bract, from Arunachal Pradesh. The new species shares some morphological features with *H.griersonianum* R.M.Sm., *H.ellipticum* Buch.-Ham. ex Sm., *H.gomezianum* Wall. and *H.yunnanense* Gagnep. *H.griersonianum* is known so far from only its type locality in Bhutan (Fig. 1, Table 1). *H.ellipticum* is one of the widely distributed species with a large variation in its morphology. *H.gomezianum* is distributed in Myanmar, NE India and Thailand. *H.yunnanense* is native to Southwest China and Vietnam. All the aforementioned species are characterised by 1-flowered cincinnus in each bract (scarcely two flowers appearing per bract even though the whole inflorescence never completely deviates from the 1-flowered cincinnus structure).

**Table 1.** Morphological similarities and dissimilarities of *Hedychiumziroense* sp. nov. with *H.griersonianum*, *H.ellipticum*, *H.gomezianum* and *H.yunnanense*. Amongst the five species, characters that are diagnostic for *H.ziroense* sp. nov. are shown in bold.

Attributes	*Hedychiumziroense* sp. nov.	*H.griersonianum* R.M.Sm.	*H.ellipticum* Buch.-Ham. ex Sm.	*H.gomezianum* Wall.	*H.yunnanense* Gagnep.
Literature source for morphological characters	This paper	Smith (1991, 1994)	Smith (1811), Sanoj (2011)	Wallich (1853), Sirirugsa (1991), Sanoj (2011), Wongsuwan and Picheansoonthon (2011)	Gagnepain (1907), Wu and Larsen (2000), MNHN (2018)
**Plant height (m)**	1.6	1	0.6–1.1	0.5–1.2	0.5–1.0
**Lamina size (cm)**	35–60 × 13–17	30–35 × 8–10	24–39 × 7–15	34–37 × 5.5–7.5	20–40 × 10
Inflorescence type	cylindrical	elongate spike; elliptical	elliptical	cylindrical	cylindrical
Inflorescence density	dense	dense	dense	lax	lax
Inflorescence length (cm)	16	12	4–10	5–20	15–20
Bract type	folded; involute	imbricating	imbricating	folded; involute	folded; involute
Bract shape	elliptic	oblong	elliptic	elliptic	lanceolate
Bract indumentum (adaxial)	absent	absent	absent	present	absent
**Bract size (cm)**	4.0–4.5 × 1.0–1.2	1.0–2.0 × 0.5–0.8	1.5–3.2 × 1.0–3.0	2.8–3.1 × 0.9–1.1	1.5–2.5
Bracteole size (cm)	1.0–1.5	1.0	0.9–1.2	1.0–1.3	1.2–1.8
**Number of flowers per cincinnus**	2–3	1	1	1	1
Calyx length (cm)	2.8–3.2	2.0–2.5	1.7–3.2	2.7–3.0	1.7–2.8
Floral tube length (cm)	4.7–5.0	3.5–4.0	4.0–7.0	3.0–4.8	3.5–5.0
Corolla lobe length (cm)	4.0–4.5	2.5–3.5	2.8–5.6	4.0–4.5	2.5–3.0
Lateral staminode shape	narrowly oblanceolate	narrowly elliptic	spatulate	narrowly oblanceolate	oblong-linear
Lateral staminode length (cm)	3.5–4.0	1.5–1.8	2.5–5.0	3.1–3.2	2.2–2.9
Labellum shape	elliptic	elliptic	spatulate	oblanceolate	obovate
Labellum length (cm)	3.5–4.0	1.0–1.3	2.1–4.0	2.6–2.7	2.0–2.5
Labellum sinus depth	½ or more the length of labellum	⅓ the length of labellum	less than ⅓ the length of labellum	less than ½ the length of labellum	½ the length of labellum
Filament length (cm)	5.5–6.0	2.5–3.0	4.0–7.0	3.5–4.0	3.5–4.2
**Anther colour**	orange	crimson	orange-red	red	red
Anther length (cm)	0.9–1.2	0.6–1.0	1.6–1.8	1.0–1.5	1.0–1.2
Flowering	August-September	June-July	June-August	July-August	July-September
Elevation (m)	>1700	1100	305–2440	500–1700	2000–3000
Distribution	Arunachal Pradesh	Bhutan	Nepal, Bhutan, NE India, China, Myanmar	NE India, Myanmar, Thailand	China, Vietnam

## Materials and methods

Field collection trips were planned and conducted in August and September 2017 in selected regions within the state of Arunachal Pradesh. Metadata collection included scoring of morphological, phenological as well as ecological characters. Morphological measurements were taken using both ruler and digital calipers. Collections included herbarium and spirit samples and leaf tissues in silica for further molecular studies.

All *Hedychium* protologues were examined including monographs and revisions; Koenig 1783, Smith 1811, Roscoe 1828, Wallich 1853, Horaninow 1862, Baker 1892, Schumann 1904, Naik and Panigrahi 1961, Rao and Verma 1969, Srivastava 1984, Smith 1991, Sirirugsa and Larsen 1995, Wu and Larsen 2000, Sanoj 2011, Wongsuwan and Picheansoonthon 2011, Thomas 2015). Herbarium collections, including type specimens, were consulted at ARUN, ASSAM, BHPL, BO, BSA, BSHC, BSI, CAL, MH, QBG, SING, TBGT and in online databases (Global Plants: https://plants.jstor.org/; Kew Herbarium Catalogue: http://apps.kew.org/herbcat/; Muséum national d’Histoire naturelle: https://science.mnhn.fr/; Smithsonian Institution: https://www.si.edu/; The Linnean Collections: http://linnean-online.org/; Zingiberaceae Resource Centre: http://padme.rbge.org.uk/ZRC/; see Appendix 1).

The conservation status for the new species was evaluated according to the International Union for Conservation of Nature (IUCN) guidelines (2017).

## Taxonomic treatment

### Hedychium ziroense

Taxon classificationPlantaeZingiberalesZingiberaceae

V.Gowda & Ashokan sp. nov.

urn:lsid:ipni.org:names:60478024-2

Fig. 2

#### Diagnosis.

**Figure 2.**
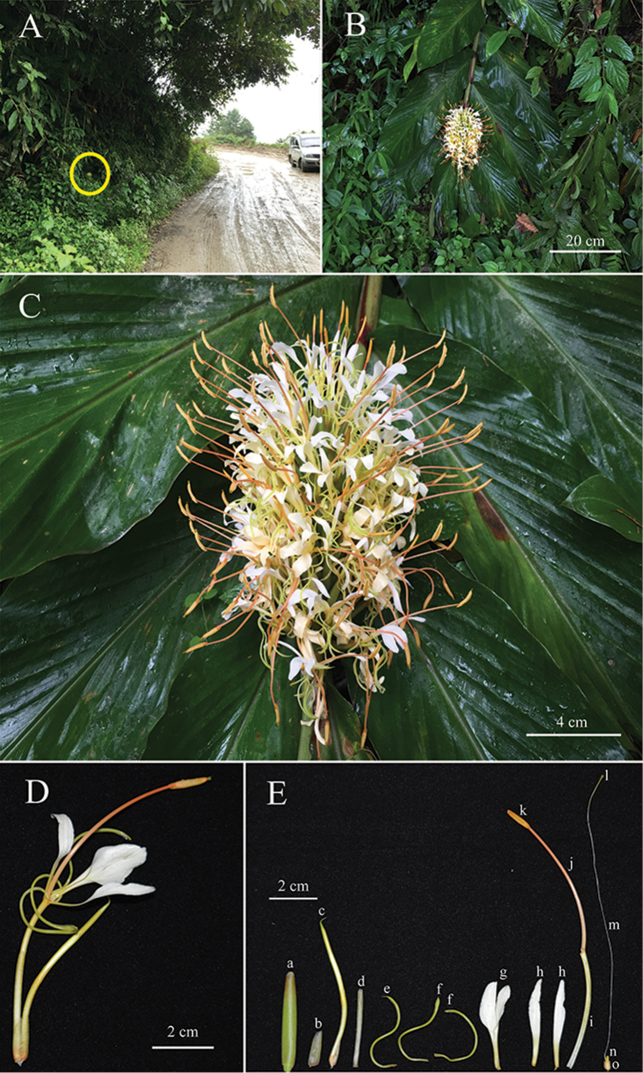
*Hedychiumziroense* V.Gowda & Ashokan, sp. nov. **A** Habitat **B** Habit **C** Inflorescence **D** Flower **E** Floral dissection **a** Bract **b** Bracteole **c** Unopened bud **d** Calyx **e** Dorsal corolla lobe **f** Lateral corolla lobe × 2 **g** Labellum **h** Lateral staminode × 2 **i** Floral tube **j** Filament **k** Anther **l** Stigma **m** Style **n** Epigynous nectary × 2 **o** Ovary. Photographed by Ajith Ashokan.

**Figure 3.**
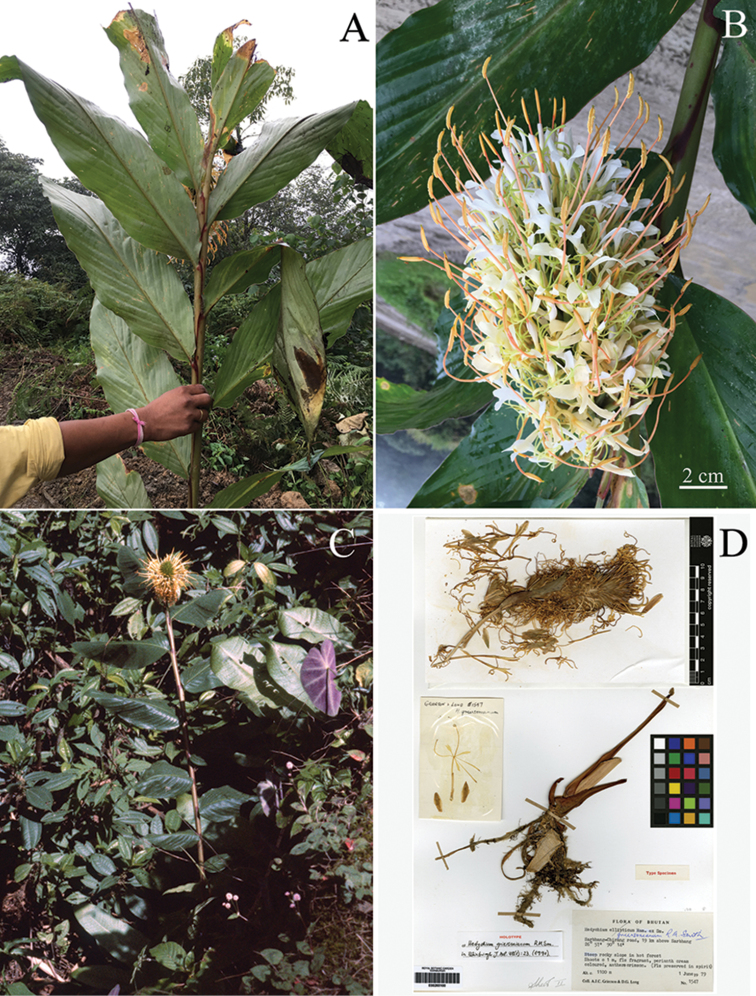
Comparison of *Hedychiumziroense* and *H.griersonianum*. **A, B** Shoot and inflorescence of *H.ziroense* sp. nov. **C***H.griersonianum***D** Holotype of *H.griersonianum* R.M.Sm. Photo Credits: **A, B** Ajith Ashokan **C** Andrew Grierson. © Royal Botanic Garden Edinburgh 2018; **D**http://data.rbge.org.uk/herb/E00265100 © Royal Botanic Garden Edinburgh 2018.

**Figure 4.**
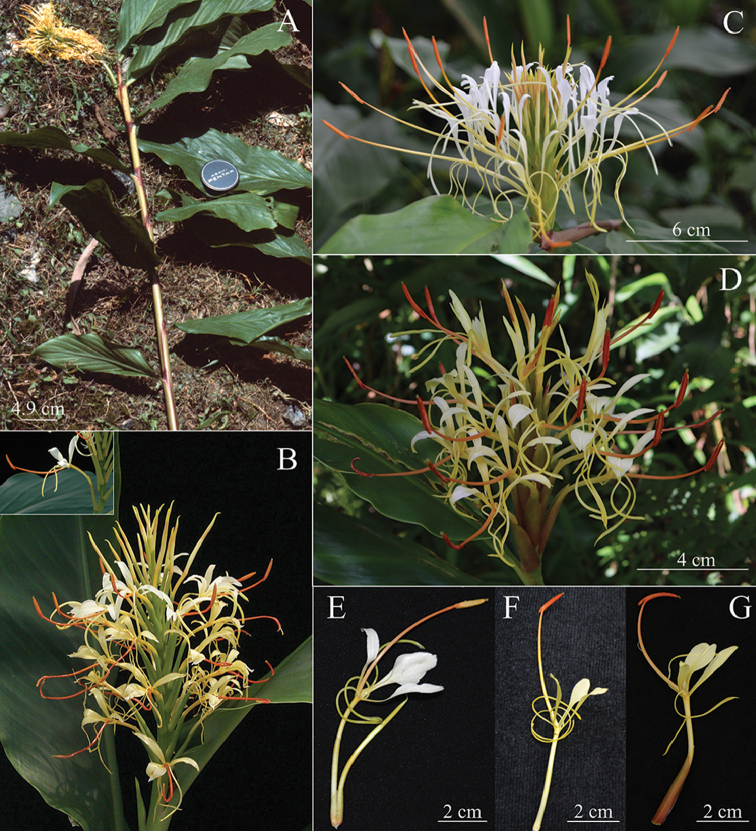
**A***Hedychiumgriersonianum***B** Inflorescence of *H.yunnanense* (flower in the inset) **C** Inflorescence of *H.ellipticum***D** Inflorescence of *H.gomezianum***E** Flower of *H.ziroense***F** Flower of *H.ellipticum***G** Flower of *H.gomezianum*. Photo Credits: **A** Andrew Grierson. © Royal Botanic Garden Edinburgh 2018; **B** Leslie Brothers. © United States National Herbarium (US); **C, E.** Ajith Ashokan; **D, F, G** Preeti Saryan.

Based on inflorescence shape and floral characters such as flower colour, relative length of filament to the labellum, relative length of corolla lobes to lateral staminodes and labellum, *Hedychiumziroense* V.Gowda & Ashokan, sp. nov. is morphologically similar to *H.griersonianum* R.M.Sm., *H.ellipticum* Buch.-Ham. ex Sm., *H.gomezianum* Wall. and *H.yunnanense* Gagnep., but it can be easily distinguished from the aforementioned species by lamina length (up to 60 cm long in *H.ziroense* vs. 40 cm or less in *H.griersonianum*, *H.ellipticum*, *H.gomezianum* and *H.yunnanense*), bract length (4 cm or more in *H.ziroense* and less than 4 cm in *H.griersonianum*, *H.ellipticum*, *H.gomezianum* and *H.yunnanense*), number of flowers per cincinnus (2-3 flowered in *H.ziroense* vs. 1-flowered in *H.griersonianum*, *H.ellipticum*, *H.gomezianum* and *H.yunnanense*), relative lengths of bract and calyx (bract always longer than calyx in *H.ziroense* vs. bract length equal or less compared to calyx in *H.griersonianum*, *H.ellipticum*, *H.gomezianum* and *H.yunnanense*) and anther colour (orange in *H.ziroense* vs. crimson in *H.griersonianum*, orange-red in *H.ellipticum*, red in both *H.gomezianum* and *H.yunnanense*), (Figs 2, 3 and 4; Table 1).

#### Type.

**INDIA.** Arunachal Pradesh, Lower Subansiri District, about 5 km before Ziro town along the Itanagar-Ziro road. Terrestrial herbs on the edge of sub-tropical broad-leaved evergreen forest, 27°32'13.19"N, 93°47'35.70"E, 1717 m a.s.l., A. Ashokan, VG2017-AR1989, 08 September 2017 (flowering). Holotype: BHPL!; isotypes: ARUN!, BHPL!, CAL!. Fig. 2.

#### Description.

Terrestrial, rhizomatous herb up to 1.6 m tall; rhizome branching parallel to leaf distichy, pale brown externally, creamy white internally; leafy shoot slanting with an erect inflorescence; basal sheath pinkish-red. Leaves subsessile; sheath purplish-red; ligule 1.8–2.3 cm wide, ovate, glabrous, purplish-red; lamina 35–60 × 13–17 cm, elliptic-lanceolate, glabrous, dark green (adaxial) and pale green (abaxial); acumen 1.5–3.0 cm long, ends in a small caducous appendage; base rounded, red protrusion at the junction of ligule and leaf base. Inflorescence 16 × 8 cm, dense-spike, erect, cylindrical; rachis pubescent, remains more hidden due to overcrowding of bracts and flowers; bracts 4.0–4.5 × 1.0–1.2 cm, elliptic, glabrous, green, tinged with red, folded, involute, 2–3 flowered; bracteoles 1.0–1.5 cm long, tubular, pubescent, greenish-red. Flowers 11.5–12.5 cm long, ascending, white (when fresh), turn creamy white the next day, many flowers open simultaneously, mildly fragrant in the daytime and strongly fragrant at night; calyx 2.8–3.2 cm long, tubular, translucent, 3-toothed, slightly hairy at tip; floral tube 4.7–5.0 cm long, glabrous, curved, greenish-yellow; corolla lobes linear, curled, orientated downwards, glabrous, light green; dorsal lobe 4.2–4.5 cm long, linear, curled, glabrous, beaked at tip, embracing the filament and anther in the bud stage, light green; lateral lobes 4.0–4.3 cm long, linear, curled (single curl), non-beaked at tip, glabrous, light green; lateral staminodes 3.5–4.0 cm long, widest part 0.5 cm, narrowly oblanceolate, one edge linear, obtuse, unguiculate, upper halves reflexed backwards, positioned acute (< 70°) with respect to labellum, white, light yellow towards base; labellum 3.5–4.0 × 1.0–1.5 cm, elliptic, glabrous, white with light yellow blotch, deeply bilobed, canaliculate, gradually clawed; lobes acute, upper halves reflexed backwards; sinus 1.5–2 cm deep; claw 1.0–1.3 × 0.2–0.3 cm; filament 5.5–6.0 cm long, slightly arching, deeply grooved on one side, pale orange, bright orange towards tip; anther 0.9–1.2 cm long, linear, basifixed, orange; thecae 2, split longitudinally; connective 0.1–0.2 cm long, bright orange; stigma 0.1 cm wide, cup-shaped, hairy at tip, exserted from the anther by at least 0.1 cm, green; style 11.5–12 cm long, filiform, white, greenish towards tip; epigynous nectaries 0.2–0.25 cm long, glabrous, yellow; ovary 0.3–0.4 × 0.2–0.4 cm, barrel-shaped, pubescent, creamy orange externally; placentation axile; ovules spherical, creamy white. Fruit not seen.

#### Distribution and habitat.

This species is known only from collection along road banks on the Itanagar-Ziro road, Lower Subansiri District, Arunachal Pradesh at an elevation of more than 1700 m.

#### Phenology and ecology.

Flowering from August to September. Flowers mildly fragrant during the day and prominently fragrant at night indicating that this species may be pollinated by nocturnal pollinators.

Sub-tropical broad-leaved evergreen forest; canopy partially shaded; soil black, clay loam; Average temperature: 25 ± 2 °C; Humidity: > 98%; Average rainfall: ~3000 mm.

Other *Hedychium* in the vicinity: *H.coccineum* Buch.-Ham. ex Sm., H.speciosumvar.gardnerianum (Ker Gawl.) Sanoj and M.Sabu, *H.stenopetalum* Lodd. and *H.wardii* C.E.C.Fisch. Except for some H.speciosumvar.gardnerianum, all other *Hedychium* were fruiting at the time of collection.

#### Etymology and vernacular name.

The specific epithet, “*ziroense*”, is derived from the type locality ‘Ziro’, the closest town to where the species was found. The town of Ziro is the headquarters of Lower Subansiri District, Arunachal Pradesh. *Ziro* is also the name of the native tribal inhabitants of the valley much before the arrival and subsequent colonisation of Apatani tribe (Ngunu Ziro pers. com.). In Apatani language, members of *Hedychium* are known by the common name “papi” (Bouchery 2016).

#### Conservation status and IUCN preliminary assessment.

At the time of collection, the area comprising this population was under threat due to the ongoing resurfacing and widening of the Ziro-Hapoli-Yazali-Kimin road. As this species is known only from the type locality, we categorise it as data deficient (DD) following the IUCN guidelines (IUCN Standards and Petitions Subcommittee 2017).

## Supplementary Material

XML Treatment for Hedychium ziroense

## Appendix 1

List of specimens examined for *Hedychiumziroense* sp. nov., *H.griersonianum*, *H.ellipticum*, *H.gomezianum* and *H.yunnanense*. Specimens from the Royal Botanic Garden Edinburgh (E), Royal Botanic Gardens, Kew (K), Linnean Society of London (LINN) and Muséum national d’Histoire naturelle (P) refer to high-resolution digital images of specimens that were examined in this study.

**Table:**

Species	Specimen(s) examined with collector and collection information.
*Hedychiumziroense* V.Gowda & Ashokan, sp. nov.	INDIA. Arunachal Pradesh, Lower Subansiri District, about 5 km before Ziro town along the Itanagar-Ziro road, 27°32'13.19"N, 93°47'35.70"E, 1717 m a.s.l., A.Ashokan VG2017-AR1989, 08 September 2017; 27°31'57.97"N, 93°47'43.35"E, 1782 m a.s.l., A.Ashokan VG2018007, 25 August 2018 (BHPL!).
*H.griersonianum* R.M.Sm.	BHUTAN. Sarbhang, 01 June 1979, A. J. C. Grierson & D. G. Long 1547 (E, K); Chukka, 03 July 1914, R. E. Cooper 1152 (E).
*H.ellipticum* Buch.-Ham. ex Sm.	NEPAL. Narainhetty, 28 July 1902, F. Buchanan-Hamilton 8.31 (LINN); INDIA. Arunachal Pradesh, Lower Subansiri District, Jara, 17 September 1983, G. D. Pal 591 (ARUN!); Meghalaya, Khasi & Jaintia Hills, Barapani, 3500 ft., 28 June 1922, U. Kanjilal 7731 (ASSAM!); Manipur, Senapati District, Taphou Hills, 23 July 2016, N. Sarangthem VC2016c (BHPL!).
*H.gomezianum* Wall.	MYANMAR. Mergui, N. Wallich 6543 (K); THAILAND. Peninsular: Khao Khlong Yang at Khao Phra Mi, 06 July 1979, K. Larsen 30649 (E); Ranong, Pra Part Beach, 18 July 1979, C. Niyomdham 349 (E); INDIA. Arunachal Pradesh, Anjaw District, Pomagam, 1500 m., 20 October 2010, R. Gogoi 21543 (ARUN!); West Kameng District, Tenga Valley, 05 September 2017, A. Ashokan VG-AR1971 (BHPL!); Manipur, Senapati District, Taphou Hills, 01 August 2017, P. Saryan VG-MN1751 (BHPL!); Ukhrul District, 25 August 2017, A. Ashokan VG-MN1926 (BHPL!); Nagaland, Kohima, 31 August 2017, A. Ashokan VG-AR1949 (BHPL!).
*H.yunnanense* Gagnep.	CHINA. Yunnan, 24 July 1902, J. Beauvais 1284 (P); Yunnan, July 1912, G. Forrest 8708 (E); Yunnan, E. E. Maire 659 (E); VIETNAM. Dalat, 15 July 1924, F. Evrard 1039 (P); Tonkin, km 15 du col de Lô Qui Hô près Chapa, 28 July 1926, E. Poilane 12637 (P).
